# Rapid Exploration of Mixture Adsorption via Adiabatic Sampling

**DOI:** 10.1021/acs.jpcc.6c01337

**Published:** 2026-06-17

**Authors:** Caroline Desgranges, Jerome Delhommelle

**Affiliations:** † Department of Physics Applied Physics, 14710 University of Massachusetts, Lowell, Massachusetts 01854, United States; ‡ Department of Chemistry, University of Massachusetts, Lowell, Massachusetts 01854, United States

## Abstract

The efficient computational screening of large libraries of porous materials for gas storage applications relies on the ability of simulations to rapidly predict the phase diagrams of confined fluids, their adsorption properties, and the selectivity of adsorbents in separation applications. While isothermal grand-canonical simulations are commonly used to this end, their convergence rate can dramatically decrease when the thermodynamic conditions approach coexistence, giving rise to metastability and the onset of large free-energy barriers. To address this challenge, we develop an adiabatic counterpart for the simulation of mixtures, derive the acceptance rules for a grand-isochoric adiabatic Monte Carlo method, and assess its accuracy and efficiency through comparisons with isothermal grand-canonical simulations of Ar–Kr mixtures, both in the bulk and when adsorbed in an MCM-41 porous material. The results show that adiabatic simulations allow for large temperature variations, thereby resulting in the rapid sampling of the configuration space and faster convergence than the grand-canonical simulations by up to 2 orders of magnitude.

## Graphical abstract

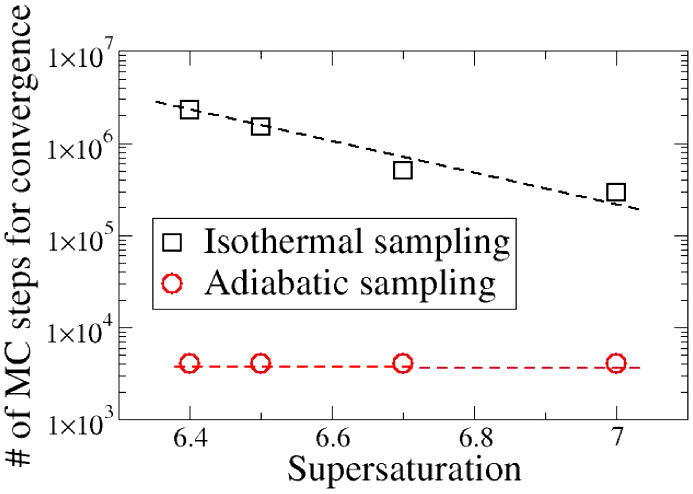

## Introduction

Metastability presents unique challenges to the determination of materials properties both experimentally and using simulations.^1^ The large free energy barriers that accompany phase transitions as conditions approach coexistence often result in a delayed onset of the transition that exceeds the time scales of conventional simulation methods.^2,3^ While enhanced sampling methods can be used to increase sampling efficacy and ensure that all configurations spanning the transition pathway are simulated^4–17^ such methods are generally computationally intensive. As a result, these advanced methods are not well suited to the automated exploration of the large combinatorial space involved in materials design and discovery such as, for instance, in the assessment of large libraries of hypothetical nanoporous materials involved in gas storage and separation processes.^18–26^ There is therefore a need for simulation methods that can rapidly determine the equilibrium state for a given system and allow for the efficient screening of materials for a specific application.

Classical nucleation theory predicts that the height of the free energy barrier is a function of the degree of supersaturation or, in other words, of how far the conditions are from equilibrium and coexistence. Temperature can often serve as a thermodynamic control parameter, since the free energy barrier of, e.g., crystal nucleation decreases when the temperature decreases below the freezing temperature.^1^ In turn, nucleation events take place much more often, and their kinetics are greatly increased, thereby alleviating any prolonged metastability and hysteresis from occurring. The same conclusion holds for different types of first-order phase transitions,^2,27–30^ with changes in temperature resulting in lower free energy barriers of nucleation both for bulk phases and under nanoconfinement.^31–40^ This prompted us in recent work^41^ to carry out simulations of single-component systems in an adiabatic statistical ensemble known as the grand-isochoric adiabatic ensemble,^42–44^ and denoted by the set of thermodynamic variables (μ, *V*, *L*), where μ is the chemical potential, *V* the volume of the system, and *L* the Hill energy. In this adiabatic ensemble, the heat function is the fixed quantity (here, the Hill energy *L*) and the temperature is allowed to vary during sampling.^43,45–49^ The system thus has the ability to adapt its temperature while undergoing a phase transition^50–53^ leading to a much faster convergence of the simulations toward equilibrium.^41^

In this work, we turn to the case of mixtures, as mixture adsorption is central to many practical applications.^54–68^ It is, for instance, the case in separation processes where adsorbents adsorb selectively mixture components.^69–76^ Similarly to the adsorption of single-component systems, mixture adsorption gives rise to metastability.^77–80^ The development of efficient simulation methods that can rapidly converge toward the equilibrium loading curve and adsorption isotherms is thus of paramount importance. This need is even more pressing when considering the additional dimensions to be sampled for a multicomponent mixture. While the idea of an adiabatic counterpart to the well-established grand-canonical simulations had been proposed more than 30 years ago, there have been comparatively very few applications of this simulation approach. Previous work was able to validate the approach for simple, single-component, systems through comparisons of adiabatic simulation results with results obtained with the more conventional isothermal simulation methods. We posit that the recently identified ability of grand-isochoric adiabatic simulations to reliably and efficiently overcome metastability and bimodality in simulations of adsorption processes provides a promising incentive to further develop this method to handle multicomponent systems of molecular compounds. This requires, however, developing the underlying mathematical formalism, derive acceptance rules for the simulation algorithm in a Monte Carlo framework, and carrying out a thorough assessment of the simulation method. We begin here with the case of mixtures, validating the approach on the case of a mixture of atomic gases, and will focus in upcoming work or extending the approach to the adsorption of molecular compounds and their mixtures. To our knowledge, previous work on the grand-isochoric adiabatic ensemble has only considered single-component systems.^53,81–86^ The first goal of this work will thus consist of extending the formalism to multicomponent systems and determining the acceptance rules for its implementation as a simulation method in a Monte Carlo framework. The results obtained with this new approach will then be assessed through a detailed comparison with results obtained with the widely used isothermal grand-canonical simulations for Ar–Kr mixtures. The second goal of this work will consist of establishing that adiabatic simulations allow for faster convergence than their isothermal counterparts. To this end, we will examine how grand-isochoric adiabatic enable a rapid exploration of the configuration space by allowing for temperature to vary during the phase transitions occurring in bulk Ar–Kr mixtures. On the other hand, grand-canonical simulations keep temperature constant and are therefore susceptible to free energy barriers and metastability. We further demonstrate that the same conclusions hold during mixture adsorption in nanocapillaries.

The paper is organized as follows. We start with the development of the grand-isochoric adiabatic simulation method for mixtures and derive the acceptance rules to implement these simulations within a Monte Carlo framework. We then present the *ab initio* many-body model used for Argon–Krypton mixtures, the model used for the silica mesoporous MCM-41 adsorbent, and the technical details for the simulations. Before delving into the comparison between adiabatic and isothermal simulations, we determine how the heat function (Hill energy in the case of the grand-isochoric ensemble) varies along phase boundaries for the Ar–Kr mixture and thus identify the locus for phase coexistence locus in terms of the Hill energy using data from previous experimental and simulation work. We proceed to validate the grand-isochoric adiabatic simulation method through a careful comparison with grand-canonical simulation results for Ar–Kr mixtures. We then show how adiabatic simulations enable faster convergence in bulk systems and for the adsorption of mixtures in MCM-41 as the conditions approach coexistence, before drawing the main conclusions from this work in the last section.

## Methods

### Grand-Isochoric Adiabatic Ensemble

We start with a brief summary of the key concepts underlying adiabatic statistical ensembles and, especially, the grand-isochoric adiabatic ensemble for a single-component system. Following pioneering work by Guggenheim^87^ and Hill^44^ Graben and Ray^46^ discussed how eight different statistical ensembles could be defined for a single-component system in the absence of any external field. These eight ensembles are organized in pairs of isothermal and adiabatic ensembles, with the former remaining at a constant temperature through heat exchanges between the system and the reservoir, and the latter being thermally insulated from their surroundings.^43,45^ Within an isothermal-adiabatic pair of ensembles, the thermodynamic potentials are related to each other by Legendre transforms.^48^ The most-well-known pair of ensembles is the canonical-microcanonical pair for which we have *A*(*N*, *V*, *T*) = *E* – *TS*(*N*, *V*, *E*). Here, the Helmholtz free energy *A*(*N*, *V*, *T*) is the thermodynamic potential for the (isothermal) canonical (*N*, *V*, *T*) ensemble and the entropy *S*(*N*, *V*, *E*) is the thermodynamic potential for the (adiabatic) microcanonical (*N*, *V*, *E*) ensemble.

If the volume of the system *V* is kept fixed and the number of atoms *N* is now allowed to fluctuate, the isothermal ensemble becomes the grand-canonical (μ, *V*, *T*) ensemble and its adiabatic counterpart is the grand-isochoric adiabatic (μ, *V*, *L*) ensemble. Here, the heat function *L* denotes the Hill energy, defined as *L* = *E* – *μN*. For this pair of ensembles, the relation between thermodynamic potentials is given by

1 J(μ,V,T)=L−TS(μ,V,L)

in which functions *J*(μ, *V*, *T*) and *S*(μ, *V*, *T*) are the thermodynamic potentials for the (μ, *V*, *T*) and (μ, *V*, *L*) ensembles, respectively. Interestingly, entropy plays the role of thermodynamic potential for all adiabatic ensembles,^43,85,88–91^ Its relation to the phase space volume Ω­(μ, *V*, *L*) is given by

2 $$S=k_{B}\;\log\Omega \left(\mu ,V,L\right)$$

Alternatively, entropy can also be defined using the density of states ω instead of the phase space volume Ω, with both entropy definitions leading to results that are equivalent in the thermodynamic limit.^91,92^ The phase space volume Ω­(μ, *V*, *L*) is defined by a multidimensional integral as

3 $$\Omega \left(\mu ,V,L\right)=\overset{\infty }{\underset{N=0}{\sum }}\frac{1}{N!h^{3N}}\int ...\int \Theta \left(L+\mu N-H\right)dr^{3N}dp^{3N}$$

where Θ denotes the Heaviside function, *d* **r** ^3*N*^ and *d* **p** ^3*N*^ the 3*N* position and momenta coordinates, respectively, for the *N* atoms of the system, and H=K+U the Hamiltonian of the system. Performing the integration over the momenta and making the variable change *q*_*i*_ = *r*_*i*_/V^1/3^ so that the position coordinates become dimensionless^85,89,93^ we find for the phase space volume Ω­(μ, *V, L*) and for the density of states ω­(μ, *V*, *L*) the following set of equations

4 $$\{\begin{array}{l} \Omega \left(\mu ,V,L\right)=\overset{\infty }{\underset{N=0}{\sum }}\frac{(2\pi m{)}^{3N/2}V^{N}}{N!h^{3N}\Gamma \left(\frac{3N}{2}+1\right)}\int ...\int {\left(L+\mu N-U\right)}^{3N/2}\Theta \left(L+\mu N-U\right)dq^{3N}\\ \omega \left(\mu ,V,L\right)=\overset{\infty }{\underset{N=0}{\sum }}\frac{(2\pi m{)}^{3N/2}V^{N}}{N!h^{3N}\Gamma \left(\frac{3N}{2}\right)}\int ...\int {\left(L+\mu N-U\right)}^{3N/2-1}\Theta \left(L+\mu N-U\right)dq^{3N} \end{array}$$

where the density of states ω­(μ, *V*, *L*) is obtained by differentiating Ω­(μ, *V*, *L*) with respect to *L*. In both integrals, the kinetic energy of the system *K* = (*L* + *μN* – *U*) appears as a multiplying factor and as the argument of the Heaviside function. The weight factors for the Metropolis acceptance rules^85,89,94^ can be obtained from the equation for ω­(μ, *V*, *L*) (second equation in eq 4). The acceptance rule for a generic move from an “old” configuration, denoted by *o* and associated with a number of atoms *N*_*o*_, a potential energy *U*_*o*_, and a weight factor *W*_*o*_, to a “new” configuration, denoted by *n* and associated with with a number of atoms *N*_*n*_, a potential energy *U*_*n*_, and a weight factor *W*_*n*_, is given by

5 $$acc\left(o\to n\right)=\left(1,\frac{W_{n}}{W_{o}}\right)$$

where, for instance, the weight factor *W*_*o*_ for the “old” configuration is

6 $$W_{o}=\frac{{\left(2\pi m\right)}^{3N_{o}/2}}{N_{o}!h^{3N_{o}}\Gamma \left(\frac{3N_{o}}{2}\right)}V^{N_{o}}{\left(L+\mu N_{o}-U_{o}\right)}^{3N_{o}/2-1}$$

### Extension to Mixtures

We now extend the grand-isochoric adiabatic ensemble to multicomponent systems using the Ar–Kr binary mixture as an example (Ar = 1, Kr = 2). This leads to two main changes in the equations. The first change takes place in the “ideal gas” multiplying factor that appears before the integrals in eq 4 and arises from performing the integral over the momenta coordinates for the mixture. The change in the multiplying factor is very general. For simplicity, we show below its expression for an ideal mixture with *N*_1_ atoms of mass *m*_1_ and *N*_2_ atoms of mass *m*_2_ for each component (*i* = 1, 2) in the microcanonical ensemble. In this case, the phase space volume Ω­(*N*_1_, *N*_2_, *V*, *E*) is given by

7 $$\Omega \left(N_{1},N_{2},V,E\right)=V^{N_{1}+N_{2}}\int ...\int \overset{3N_{1}}{\underset{i}{\prod }}dp_{i}\overset{3N_{2}}{\underset{j}{\prod }}dp_{j}$$

for sets of momenta such that $\overset{3N_{1}}{\underset{i=1}{\sum }}\frac{p_{i}^{2}}{2m_{i}}+\overset{3N_{2}}{\underset{j=1}{\sum }}\frac{p_{j}^{2}}{2m_{j}}\leq E$ and a system’s Hamiltonian defined as $H=\overset{3N_{1}}{\underset{i=1}{\sum }}\frac{p_{i}^{2}}{2m_{1}}+\overset{3N_{2}}{\underset{j=1}{\sum }}\frac{p_{j}^{2}}{2m_{2}}$ . To solve eq 7, we use Dirichlet’s integral formula.^95^ According to this formula, we have for a given integral I

8 $$\begin{array}{ll} I & =\int ...\int t_{1}^{\alpha_{1}-1}t_{2}^{\alpha_{2}-1}...t_{n}^{\alpha_{n}-1}dt_{1}dt_{2}...dt_{n}\\ & =\frac{b_{1}^{\alpha_{1}}b_{2}^{\alpha_{2}}...b_{n}^{\alpha_{n}}}{\beta_{1}\beta_{2}...\beta_{n}}\times \frac{\Gamma \left(\alpha_{1}/\beta_{1}\right)\Gamma \left(\alpha_{2}/\beta_{2}\right)...\Gamma \left(\alpha_{n}/\beta_{n}\right)}{\Gamma \left(\alpha_{1}/\beta_{1}+\alpha_{2}/\beta_{2}+...+\alpha_{n}/\beta_{n}+1\right)} \end{array}$$

in which *t*_*i*_, *b*_*i*_, *β*_*i*_ are positive and satisfy the following condition

9 $${\left(t_{1}/b_{1}\right)}^{\beta_{1}}+{\left(t_{2}/b_{2}\right)}^{\beta_{2}}+...+{\left(t_{n}/b_{n}\right)}^{\beta_{n}}\leq 1$$

If we choose the following values for the Dirichlet parameters *α*_*k*_ = 1, *t*_*k*_ = *p*_*i*_, *β*_*k*_ = 2, *b*_*k*_ = (2*m*_1_ *E*)^1/2^, *k* = 1, 2, ···, 3*N*_1_, α_*k*_ = 1, *t*_*k*_ = *p*_*j*_, *β*_*k*_ = 2, *b*_*k*_ = (2*m*_2_ *E*)^1/2^, *k* = 3*N*_1_ + 1, ···, 3*N*_1_ + 3*N*_2_, we obtain for the phase space volume

10 $$\Omega \left(N_{1},N_{2},V,E\right)=\frac{V^{N_{1}+N_{2}}{\left(2\pi m_{1}\right)}^{3N_{1}/2}{\left(2\pi m_{2}\right)}^{3N_{2}/2}}{h^{3(N_{1}+N_{2})}N_{1}!N_{2}!\Gamma \left[3\left(N_{1}+N_{2}\right)/2+1\right]}E^{3(N_{1}+N_{2})/2}$$

The change in the multiplying factor of eq 4 for the grand-isochoric ensemble can thus be summarized as follows

11 $$\frac{{\left(2\pi m\right)}^{3N/2}V^{N}}{h^{3N}N!\Gamma \left(\frac{3N}{2}+1\right)}\to \frac{{\left(2\pi m_{1}\right)}^{3N_{1}/2}{\left(2\pi m_{2}\right)}^{3N_{2}/2}V^{N_{1}+N_{2}}}{h^{3(N_{1}+N_{2})}N_{1}!N_{2}!\Gamma \left[3\left(N_{1}+N_{2}\right)/2+1\right]}$$

The second change takes place in the expression for the kinetic energy *K* in eq 4 for the grand-isochoric ensemble and is given by

12 $$\left(L+\mu N-U\right)\to \left(L+\mu_{1}N_{1}+\mu_{2}N_{2}-U\right)$$

We thus obtain for the phase space volume Ω­(μ_1_, μ_2_, *V*, *L*) and for the density of states ω­(μ_1_, μ_2_, *V*, *L*) the following set of equations

13 $$\left\{ \begin{array}{ll} \Omega \left(\mu_{1},\mu_{2},V,L\right)= & \underset{N_{1}}{\sum }\underset{N_{2}}{\sum }\frac{{\left(2\pi m_{1}\right)}^{3N_{1}/2}{\left(2\pi m_{1}\right)}^{3N_{2}/2}V^{N_{1}+N_{2}}}{N_{1}!N_{2}!h^{3(N_{1}+N_{2})}\Gamma \left(\frac{3\left(N_{1}+N_{2}\right)}{2}+1\right)}\\ & \int ...\int K^{3(N_{1}+N_{2})/2}\Theta \left(K\right)dq^{3(N_{1}+N_{2})}\\ \omega \left(\mu_{1},\mu_{2},V,L\right)= & \underset{N_{1}}{\sum }\underset{N_{2}}{\sum }\frac{{\left(2\pi m_{1}\right)}^{3N_{1}/2}{\left(2\pi m_{2}\right)}^{3N_{2}/2}V^{N_{1}+N_{2}}}{N_{1}!N_{2}!h^{3(N_{1}+N_{2})}\Gamma \left(\frac{3\left(N_{1}+N_{2}\right)}{2}\right)}\\ & \int ...\int K^{3(N_{1}+N_{2})/2-1}\Theta \left(K\right)dq^{3(N_{1}+N_{2})} \end{array}\right.$$

where *K* = *L* + μ_1_ *N*_1_ + μ_2_ *N*_2_ – *U*.

### Acceptance Rules for Grand-Isochoric Adiabatic Simulations of Mixtures

The equation for ω­(μ_1_, μ_2_, *V*, *L*) in eq 13 allows for the determination of the acceptance rules for the grand-isochoric adiabatic ensemble. If we denote by *b*_*i*_ for *i* = 1, 2 the ratio (2π*m*_*i*_/*h* ^2^)^3/2^, the general acceptance rule for a Monte Carlo (MC) move in the grand-isochoric adiabatic ensemble from an “old” configuration *o* with, for the number of atoms, the set (*N*_1,*o*_,*N*_2,o_) and a potential energy *U*_*o*_, to a “new” configuration *n* with (*N*_1,*n*_,*N*_2,n_)­and a potential energy *U*_*n*_, is given by

14 $$\begin{array}{ll} acc\left(o\to n\right) & =\min\left[1,\frac{W_{n}}{W_{o}}\right]\\ & =\min\left[1,\{{\left(b_{1}V\right)}^{N_{1,n}}{\left(b_{2}V\right)}^{N_{2,n}}N_{1,o}!N_{2,o}!\Gamma \left(3\left(N_{1,o}+N_{2,o}\right)/2\right)K_{n}^{3(N_{1,n}+N_{2,n})/2-1}\}/\{{\left(b_{1}V\right)}^{N_{1,o}}{\left(b_{2}V\right)}^{N_{2,o}}N_{1,n}!N_{2,n}!\Gamma \left(3\left(N_{1,n}+N_{2,n}\right)/2\right)K_{o}^{3(N_{1,o}+N_{2,o})/2-1}\right\}] \end{array}$$

where the kinetic energy for the “old” configuration is *K*_0_ = (*L* + *μ*_1_ *N*_1,*o*_ + μ_2_ *N*_2,o_ – *U*_*o*_) and for the “new” configuration *K*_*n*_ = (*L* + *μ*_1_ *N*_1,*n*_ + *μ*_2_ *N*_2,*n*_ – *U*_*n*_).

We consider three different types of MC moves corresponding to (i) the translation of a randomly chosen atom (either of type *i* = 1 or type *i* = 2), (ii) the insertion of an atom of either type, or (iii) the deletion of an atom of either type. For conciseness, we provide below the equations for the acceptance rules of MC moves (i)–(iii) for atom type *i* = 1 only. In the case of the translation of a randomly chosen atom of type *i* = 1, we have *N*_1,*n*_ = *N*_1,o_ = *N*_1_ and *N*_2,*n*_ = *N*_2,o_ = *N*_2_, and the acceptance rule can be written as

15 $$acc\left(o\to n\right)=\min\left[1,\frac{{\left(L+\mu_{1}N_{1}+\mu_{2}N_{2}-U_{n}\right)}^{3(N_{1}+N_{2})/2-1}}{{\left(L+\mu_{1}N_{1}+\mu_{2}N_{2}-U_{o}\right)}^{3(N_{1}+N_{2})/2-1}}\right]$$

For the insertion of an atom of type *i* = 1 at a random position, we have *N*_1,*n*_ = *N*_1,*o*_ + 1 = *N*_1_ + 1 and *N*_2,*n*_ = *N*_2,*o*_ = *N*_2_, and the acceptance rule becomes

16 $$\begin{array}{ll} acc\left(o\to n\right) & =\min[1,\frac{b_{1}V\Gamma \left(3\left(N_{1}+N_{2}\right)/2\right)}{\left(N_{1}+1\right)\Gamma \left(3\left(N_{1}+N_{2}+1\right)/2\right)}\\ & \times \frac{{[L+\mu_{1}(N_{1}+1)+\mu_{2}N_{2}-U_{n}]}^{3(N_{1}+N_{2}+1)/2-1}}{{[L+\mu_{1}N_{1}+\mu_{2}N_{2}-U_{o}]}^{3(N_{1}+N_{2})/2-1}}] \end{array}$$

For the deletion of a random chosen atom of type *i* = 1, we have *N*_1,*n*_ = *N*_1,*o*_ – 1 = *N*_1_ – 1 and *N*_2,*n*_ = *N*_2,*o*_ = *N*_2_, and the acceptance rule is

17 $$\begin{array}{ll} acc\left(o\to n\right) & =\min[1,\frac{N_{1}\Gamma \left(3\left(N_{1}+N_{2}\right)/2\right)}{b_{1}V\Gamma \left(3\left(N_{1}+N_{2}-1\right)/2\right)}\\ & \times \frac{{[L+\mu_{1}(N_{1}-1)+\mu_{2}N_{2}-U_{n}]}^{3(N_{1}+N_{2}-1)/2-1}}{{[L+\mu_{1}N_{1}+\mu_{2}N_{2}-U_{o}]}^{3(N_{1}+N_{2})/2-1}}] \end{array}$$

### Models and Simulation Protocols

#### Argon–Krypton Mixture

In this work, we use the *ab initio* potentials developed by Deiters and coworkers.^96–98^ These potentials consist of adding a three-body contribution, modeled with an Axilrod–Teller–Munroe term^99,100^ to an *ab initio* two-body potential. This approach has been shown to be highly successful for the prediction of vapor–liquid equilibria.^96–98,101–109^ Furthermore, since the parameters for the interactions between Ar and Kr atoms are also extracted from the *ab initio* calculations, this force field has the advantage of alleviating the need to use combining rules for the interactions between unlike atoms.^110–112^ To obtain the *ab initio* two-body potential, Deiters et al.,^96–98^ performed calculations at the CCSD­(T) level of theory with different correlation consistent basis sets^96–98,113^ followed by an extrapolation toward the basis set limit for the interaction energies using the 1/*X* ^3^ method, and a fit to the functional form of Korona et al.^114,115^ The resulting *ab initio* 2-body potential, which we refer to below as 2B, leads to the following equation for the interaction between two atoms *i* and *j*

18 $$\phi_{2B}\left(r_{ij}\right)=A_{ij}\exp\left[-\alpha_{ij}r_{ij}+\beta_{ij}r_{ij}^{2}\right]+\overset{5}{\underset{n=3}{\sum }}f_{2n}\left(r_{ij},B_{ij}\right)\frac{C_{2n,ij}}{r_{ij}^{2n}}$$

where *r*_*ij*_ is the distance between the two atoms *i* and *j* while *A*_*ij*_, α_*ij*_, β_*ij*_, *B*_*ij*_, *C*_2*n*,*i,j*_ are the potential parameters given in Table 1, and the *f*_2*n*_ terms are the damping functions of Tang and Toennies  eq 19

19 $$f_{2n}\left(r_{ij},b_{ij}\right)=1-\exp\left[-B_{ij}r_{ij}\right]+\overset{2n}{\underset{k=0}{\sum }}\frac{{\left(B_{ij}r_{ij}\right)}^{k}}{k!}$$

**Table 1:** Parameters for the 2-Body Interatomic Interactions (*E_H_* = 4.3597 × 10^–18^ j and  *a*_0_ = 5.29 × 10^–11^ m)

	$A/E_{h}$	$\alpha /a_{0}^{-1}$	$\beta /a_{0}^{-2}$	$B/a_{0}^{-1}$	$C_{6}/\left(E_{h}a_{0}^{6}\right)$	$C_{8}/\left(E_{h}a_{0}^{8}\right)$	$C_{10}/\left(E_{h}a_{0}^{10}\right)$
Ar–Ar	56.21	1.31938	–0.050	1.70	60.98	1941.02	62960.00
Kr–Kr	109.66	1.32512	–0.0404	1.40	120.14	3565.02	364467.00
Ar–Kr	112.08	1.42241	–0.040	1.50	94.08	2452.27	93790.30

The 2B term is then supplemented with a three-body (3B) term by adding the Axilrod–Teller–Muto (ATM) triple-dipole potential^99,100^ given below

20 $$\phi_{AT}=\sqrt[{3}]{\nu_{i}\nu_{j}\nu_{k}}\left(\frac{1+3\cos\alpha \cos\beta \cos\gamma }{r_{ij}^{3}r_{ik}^{3}r_{jk}^{3}}\right)$$

in which *r*_*ij*_, *r*_*ik*_ and *r*_*jk*_ are distances between pairs of atoms from the (*i, j, k*) triplet, α, β and γ are triangle angles, and ν_*i*_, ν_*j*_ and ν_*k*_ are Axilrod–Teller constants^97,98,116^ equal to ν_*Ar*_ = 7.32 × 10^–18^ J.Å^9^ and ν_*Kr*_ = 2.20 × 10^–17^ J.Å^9^), respectively.

Simulations of bulk mixtures are all carried out in cubic simulation cells of length *L* = 18 Å. We explicitly calculate the interactions between atoms for distances up to *L*/2 in the case of two-body interactions, and up to *L*/4 for three-body interactions, and apply long-range corrections beyond these cutoff distances.^117^

#### MCM-41 Model

We study the adsorption of Argon–Krypton mixtures in a cylindrical pore, using the same geometry and model parameters developed in previous work for the adsorption of noble gases in an MCM-41 silica mesoporous molecular sieve.^32,36,38,118,119^ The pore is placed along the *z*-axis and has a length denoted by *L*_*z*_. It is modeled with the following functional form,^32,120–123^

21 $$\begin{array}{l} U_{sf}\left(r,R\right)=\pi^{2}\rho_{s}\epsilon_{sf}\sigma_{sf}^{2}\{\frac{63}{32}{[\frac{R-r}{\sigma_{sf}}\left(1+\frac{r}{R}\right)]}^{-10}\\ \times F\left[-\frac{9}{2},-\frac{9}{2};1;{\left(\frac{r}{R}\right)}^{2}\right]-3{[\frac{R-r}{\sigma_{sf}}\left(1+\frac{r}{R}\right)]}^{-4}F\left[-\frac{3}{2},-\frac{3}{2};1;{\left(\frac{r}{R}\right)}^{2}\right]\} \end{array}$$

in which *r* is the radial coordinate of an atom (Ar or Kr) adsorbed in the pore, *R* is the pore radius with R = 17 Å in line with prior work,^32,36,38^ ρ_*s*_ is the surface density of adsorption centers and *F*(*α*, *β*; *γ*; *δ*) is the hypergeometric series. The parameters for the solid–fluid interactions are *ρ_s_ϵ*_*sf*_ = 2253 K/nm^2^ and σ_*sf*_ = 3.17 Å in the case of Argon, and *ρ_s_ϵ*_*sf*_ = 2660 K/nm^2^ and σ_*sf*_ = 3.30Å for Krypton. Interactions between atoms are calculated explicitly for distances up to 17 Å and 8.5 Å for the two-body interactions and three-body interactions, respectively, and neglect fluid–fluid interactions beyond that cutoff distance. We carry out simulations in nanopores with *L*_*z*_ = 40Å and apply the usual periodic boundary conditions along this lateral direction *z*.

#### Grand-Canonical and Grand-Isochoric Adiabatic Simulations of Mixtures

Monte Carlo (MC) simulations are performed in the grand-canonical (μ_*Ar*_, *μ*_*Kr*_, *V*, *T*) ensemble and in the grand-isochoric adiabatic (μ_*Ar*_, *μ*_*Kr*_, *V*, *L*) ensemble for Ar–Kr mixtures in the bulk and when adsorbed in MCM-41. Both simulations involve three different types of MC moves: (i) random translation of a randomly chosen Ar or Kr atom, accounting for 50% of the total number of MC moves, (ii) insertion of an additional Ar or Kr atom at a randomly selected position in the system (25% of the total number of MC moves), and (iii) deletion of a randomly selected Ar or Kr atom from the system (25% of the total number of MC moves). Since the two types of simulations employ the same sets of (μ_*Ar*_, *μ*_*Kr*_, V) values, equivalent sets of (μ_*Ar*_, *μ*_*Kr*_, *V*, *L*) and (μ_*Ar*_, *μ*_*Kr*_, *V*, *T*) simulations can be identified once we determine the correspondence between *L* and *T*. During (μ_*Ar*_, *μ*_*Kr*_, *V*, *T*) simulations, the average Hill energy ⟨*L*⟩ can be calculated as

22 $$\left\langle L\right\rangle =\left\langle U\right\rangle +\frac{3}{2}\left[\left\langle N_{Ar}\right\rangle +\left\langle N_{Kr}\right\rangle \right]k_{B}T-\mu_{Ar}\left\langle N_{Ar}\right\rangle -\mu_{Kr}\left\langle N_{Kr}\right\rangle$$

where *U* denotes the potential energy of the system and $\frac{3}{2}\left[\left\langle N_{Ar}\right\rangle +\left\langle N_{Kr}\right\rangle \right]k_{B}T$ the kinetic energy. Similarly, during (μ_*Ar*_, *μ*_*Kr*_, *V*, *L*) simulations, the average temperature of the system ⟨*T*⟩ can be computed using

23 $$\left\langle T\right\rangle =\frac{L-\left\langle U\right\rangle +\mu_{Ar}\left\langle N_{Ar}\right\rangle +\mu_{Kr}\left\langle N_{Kr}\right\rangle }{\frac{3}{2}\left[\left\langle N_{Ar}\right\rangle +\left\langle N_{Kr}\right\rangle \right]k_{B}}$$

Equations 22 and 23 thus provide a path for both simulation methods to simulate the same state point and allow for a direct validation of (μ_*Ar*_, *μ*_*Kr*_, *V*, *L*) simulations by comparison with (μ_*Ar*_, *μ*_*Kr*_, *V*, *T*) simulation results over short simulation runs for thermodynamic conditions that do not result in any hysteresis. We carry out both sets of simulations using an in-house simulation code, and the averages presented in the’Results and Discussion’ section are collected over 2 × 10^7^ MC steps.

#### Extracting Hill Energy from Available Data

For comparison purposes, we also extract from previous work the Hill energy for single-component systems and binary mixtures. Data for the single-component system were obtained from reference tables^124^ while data for the binary mixture were taken from experimental studies.^125^ We compute the molar Hill energy, denoted by L̃, along the liquid–vapor equilibrium curve for Argon using the following relation

24 L̅=TS̅−PV̅

from the available data^124^ on molar entropy S̅ and molar volume V̅ at a given coexistence point of coordinates (*P*, *T*). In addition, we use results from previous simulation work^103^ where the value taken by the grand partition function Ξ­(*μ*_*Ar*_, *μ*_*Kr*_, *V*, *T*) was computed for the Ar–Kr mixture. The molar Hill energy can then be computed from *P*, S̅, and V̅ according to

25 $$\begin{array}{c} P=V^{-1}k_{B}T\ln\Xi \left(\mu_{Ar},\mu_{Kr},V,T\right)\\ \mathrm{S̅}={[(\langle N_{Ar}\rangle +\langle N_{Kr}\rangle )k_{B}T]}^{-1}\left[k_{B}\ln\Xi \left(\mu_{Ar},\mu_{Kr},V,T\right)+E-\mu_{Ar}\left\langle N_{Ar}\right\rangle -\mu_{Kr}\left\langle N_{Kr}\right\rangle \right]\\ \mathrm{V̅}={[\langle N_{Ar}\rangle +\langle N_{Kr}\rangle ]}^{-1}V\\ \mathrm{L̅}=T\mathrm{S̅}-P\mathrm{V̅} \end{array}$$

where the internal energy *E*, the average number of atoms of each type, ⟨*N*_*Ar*_⟩ and ⟨*N*_*Kr*_⟩, and the number distribution *p*(*N*_*Ar*_, *N*_*Kr*_) are given by

26 $$\begin{array}{c} E=\underset{N_{Ar}}{\sum }\underset{N_{Kr}}{\sum }p\left(N_{Ar},N_{Kr}\right)\times \left[U\left(N_{Ar},N_{Kr}\right)+\frac{3}{2}N_{Ar}k_{B}T+\frac{3}{2}N_{Kr}k_{B}T\right]\\ \left\langle N_{Ar}\right\rangle =\underset{N_{Ar}}{\sum }\underset{N_{Kr}}{\sum }N_{Ar}\times p\left(N_{Ar},N_{Kr}\right)\\ \left\langle N_{Kr}\right\rangle =\underset{N_{Ar}}{\sum }\underset{N_{Kr}}{\sum }N_{Kr}\times p\left(N_{Ar},N_{Kr}\right)\\ p\left(N_{Ar},N_{Kr}\right)=Q\left(N_{Ar},N_{Kr},V,T\right)\exp\left[\beta \left(\mu_{Ar}N_{Ar}+\mu_{Kr}N_{Kr}\right)\right]{\Xi (\mu_{Ar},\mu_{Kr},V,T)}^{-1} \end{array}$$

## Results and Discussion

Before delving into the comparison between the adiabatic and isothermal approaches, we first discuss the locus of phase coexistence in terms of molar Hill energy which, to our knowledge, has not been discussed so far. We recall that we extract the molar Hill energy from prior simulation data^103^ and eq 25, as well as experimental data^124^ and eq 24, and provide the data in the Supporting Information.

The top panels of Figure 1 show the vapor–liquid equilibrium of a single-component Ar system modeled with the *ab initio* (+ three-body) potential we use in the *T*–ρ plane, typically obtained from isothermal simulations, and in *T*–*L* plane, spanned by adiabatic simulations, together with experimental data.^124^ The plots show excellent agreement between simulations and experiments, most notably for the molar Hill energy along the phase boundary, thereby confirming the accuracy of the potential model. We see that, for a single-component system, the phase boundaries have similar shapes in both planes, closing at the critical temperature for Argon. We also notice that the two branches are flipped, with the left low-density vapor branch on the *T*–ρ plot corresponding to the right high-*L̅* vapor branch in the *T*–*L̅* plot. We then turn to the phase diagram for the mixture and examine its projections in the *P*–*x*_*Kr*_ plane, where *x*_*Kr*_ denotes the mole fraction in Kr, and in the *P*–*L̅* plane. The graphs for the mixture are shown in the bottom panels of Figure 1. The comparison between simulation results and experimental data in the *P*–*x*_*Kr*_ plane confirms that the potentialaccurately models the *Ar* –*Kr* mixture. In the *P*–*x*_*Kr*_ plane, the phase diagram takes the shape of a closed loop with the highest pressure being reached when the system becomes pure Argon (*x*_*Kr*_ → 0) and the lowest pressure when the system becomes pure Krypton (*x*_*Kr*_ → 1). In the *L̅*–*P* plane, the phase envelope remains open with two distinct branches, with the low-*L̅* liquid branch on the left and the high-*L̅* vapor branch on the right. The end points of the *P*–*L̅* branches for the mixture match the data for single-component systems, i.e., the *L̅* of the liquid and vapor phases of Krypton for the low-pressure end and of Argon for the high-pressure end. This also confirms that the suitability of the *ab initio* (+ three-body) potential to model the variations of the Hill energy with thermodynamic conditions for the mixture.

**Figure 1:**
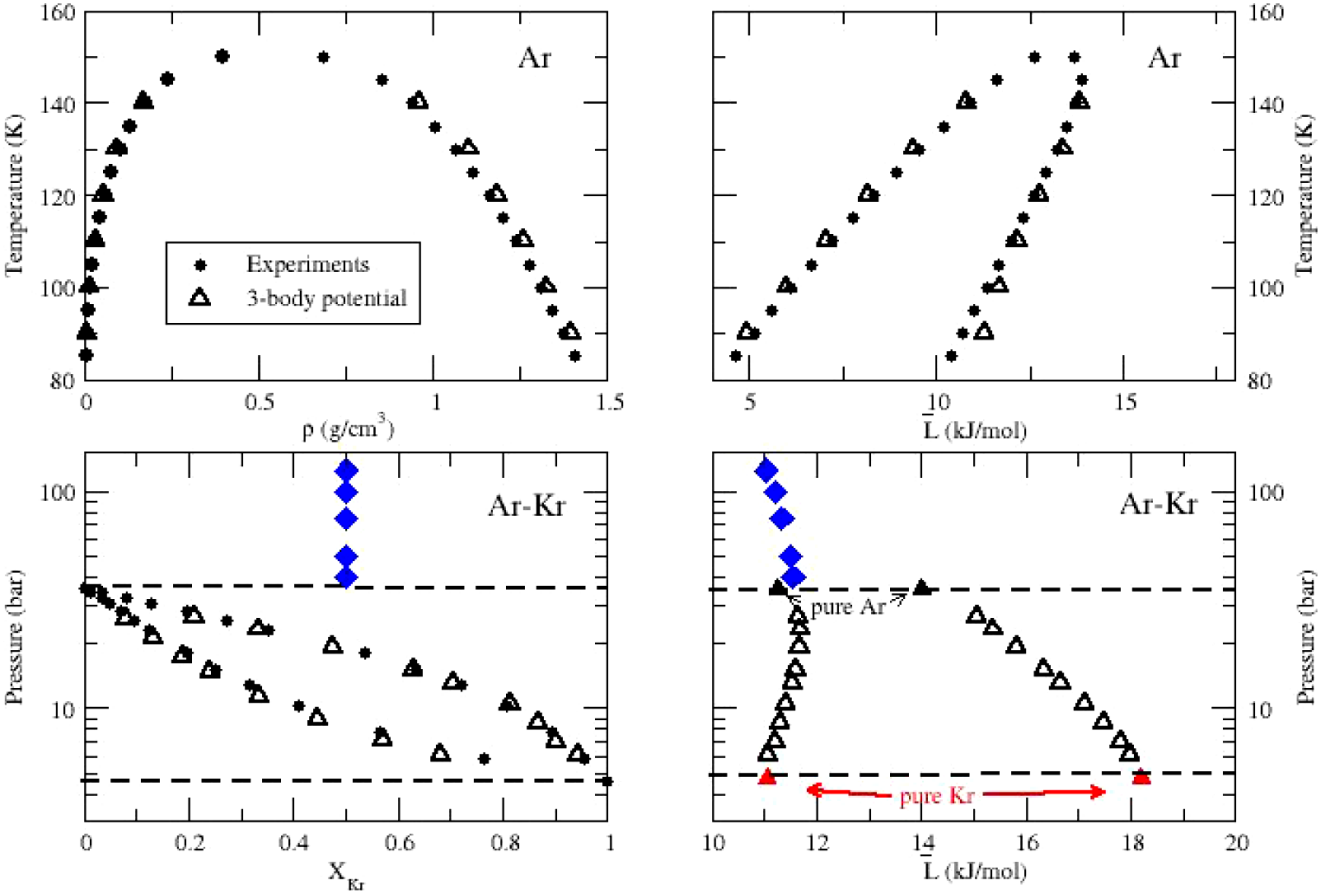
(Top panels) Phase envelope for Argon in the temperature-density plane (left) and in the temperature-molar Hill energy (*L̅*) plane (right). (Bottom panels) Phase envelope for the Argon–Krypton mixture at *T* = 143.15 K in the pressure-*x_Kr_* plane (left) and in the pressure-molar Hill energy plane (right). All plots show an excellent agreement between experimental data (filled circles) and simulation results obtained with the *ab initio*+3-body potential used in this work (open triangles). To our knowledge, phase envelopes in the *L̅*–*T* and *L̅* -P planes have not been reported previously. While the phase envelope closes at the critical point for the single-component Ar system, the phase diagram of the Ar–Kr mixture does not close. Instead, the end points for the two branches correspond to the vapor of single-component systems (Ar at high pressure, shown as filled circles, and Kr at low pressure, shown as filled triangles). We also show as filled diamonds the five liquid mixtures considered in testing the grand-isochoric adiabatic approach against the grand-canonical method.

We now focus on the validation of the grand-isochoric adiabatic approach for the simulation of mixtures. To this end, we consider a series of Ar–Kr liquid mixtures and compare the results obtained with isothermal (μ_*Ar*_, *μ*_*Kr*_, *V*, *T*) and adiabatic (μ_*Ar*_, *μ*_*Kr*_, *V*, *L*) simulations. The simulated state points are shown in the bottom plots of Figure 1 and correspond to equimolar liquid mixtures with *P* ranging from 40 bars to 125 bars. The simulation runs are carried out under equivalent conditions, i.e., with chemical potentials and molar Hill energy extracted from previous work^103^ using eq 25. The simulation conditions are summarized in Table 2, together with the results obtained for the molar Hill energy *L̅* and internal energy *E* with (grand-canonical) isothermal and grand-isochoric adiabatic simulations. We draw the attention on the fact that the input parameter in the adiabatic simulations is the extensive value of *L* and thus provide in the table the simulation parameter used as input (here given in Hartree for consistency with the *ab initio* potential parameters). To compare the thermodynamic properties obtained with the two types of simulations, we also report the corresponding intensive property, the molar Hill free energy *L̅*. The results obtained for *L̅* with the two approaches (see Table 2) are in very good agreement. We expand the comparison to include the variations in the temperature of the system, Ar mole fraction, as well as the potential energy and the total number of atoms in the system in Figure 2. The plots show a very good agreement between the results obtained with the two methods for all properties, which holds across the range of pressures. This allows us to validate the (μ_*Ar*_, *μ*_*Kr*_, *V*, *L*) approach for mixture property prediction.

**Table 2:** Equimolar Ar–Kr Mixture at *T* = 143.15 K and *P* Rsanging from 40 to 125 bars^a^

*P* (bar)	μ_*Ar*_ (kJ/mol)	μ_*Kr*_ (kJ/mol)	*L* (*E*_*H*_)	*L̅*_*A*_ (kJ/mol)	*L̅*_*I*_ (kJ/mol)	*E̅*_*A*_ (kJ/mol)	*E̅*_*I*_ (kJ/mol)
40	–13.86	–17.27	2.116	11.53	11.54	–4.04	–4.03
50	–13.81	–17.23	2.117	11.46	11.48	–4.07	–4.05
75	–13.72	–17.15	2.112	11.31	11.33	–4.13	–4.11
100	–13.62	–17.07	2.106	11.19	11.20	–4.16	–4.14
125	–13.53	–16.98	2.098	11.03	11.03	–4.23	–4.25

a For each *P*, we provide the chemical potential used in the simulations for argon (μ_Ar_), krypton (μ_*kr*_, *V*, *L*) , and the extensive value for *L* (in Hartree) used as input parameter for adiabatic simulations. We also report simulation results obtained for the molar hill energy (*L̅*) and the internal energy (*E*). Adiabatic simulation results are denoted by an *A* subscript, while isothermal simulation results are denoted by an *I* subscript (uncertainties are of the order of the last reported digit).

**Figure 2:**
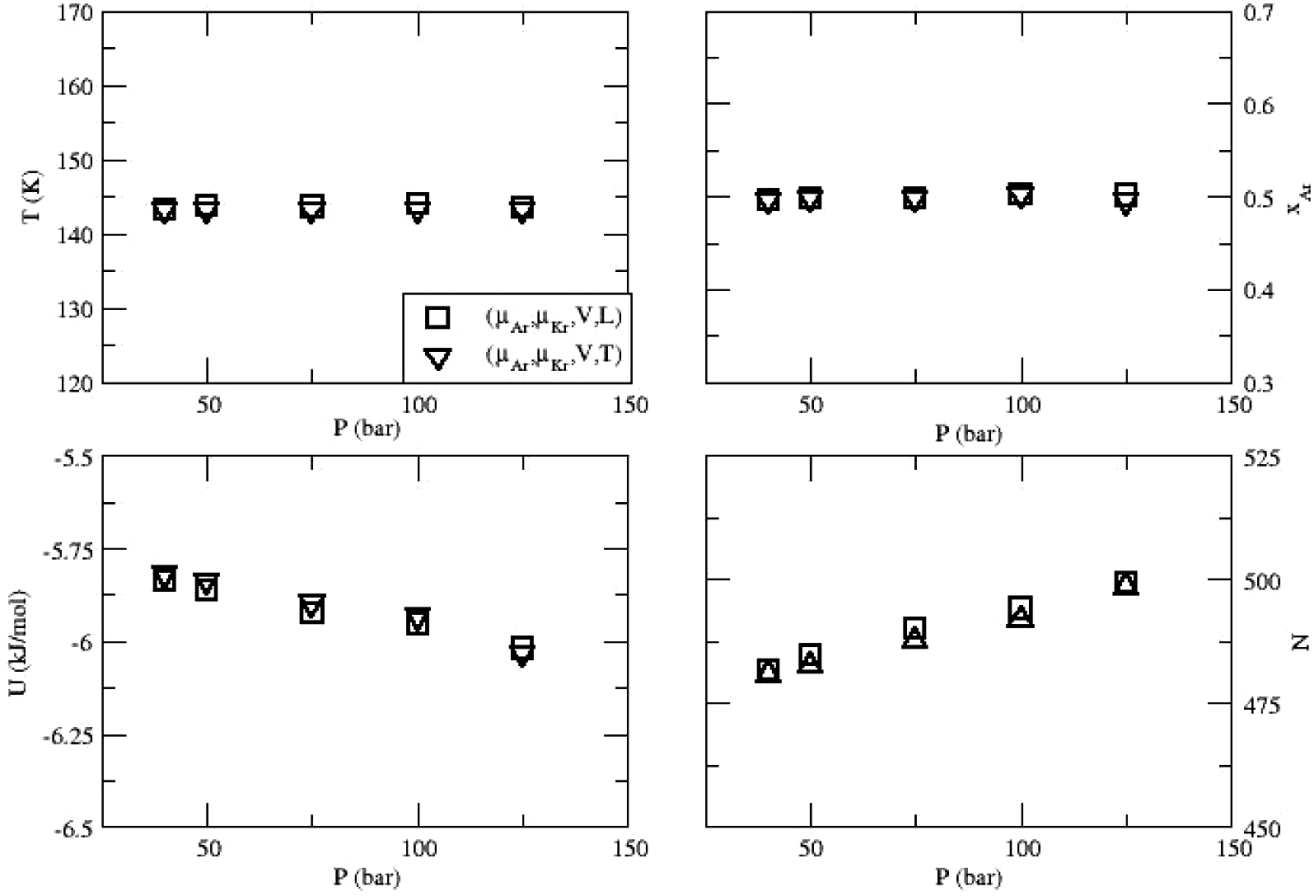
Argon–Krypton mixture for *x_Kr_* = 0.5 and *T* = 143.15 K. Plots of *T* vs *P* (top left), *x_Ar_* vs *P* (top right), potential energy (*U*) vs *P* (bottom left), and total number of atoms (*N*) vs *P* (bottom right). Average properties are collected over runs of 2 × 10^7^ MC steps during (μ*_Ar_*, *μ_Kr_*, *V*, *L*) (open squares) and (μ*_Ar_*, *μ_Kr_*, *V*, *T*) (open triangles) simulations, showing that adiabatic and isothermal simulations provide results in very good agreement with each other.

We have used Hill energy data extracted from previous work so far. If we now wish to carry out adiabatic simulations at thermodynamic conditions for which the Hill energy is not yet known, how do we determine its value? To better understand how the total Hill energy varies with temperature, we run adiabatic simulations with a gradually increasing *L* value, while keeping (μ_*Ar*_, *μ*_*Kr*_) fixed. We show the results in Figure 3 (see Supporting Information for numerical values). The average temperature *T*_*A*_, calculated using eq 23 and collected during the adiabatic simulations, is shown in the left panel of Figure 3 for increasing *L* values. We also show the (*T*, *L*_*I*_) point obtained during the isothermal (grand-canonical) simulation for the state point corresponding to *P* = 40 bar and *T* = 143.15 K. Around the target value of 143.15 K, *L*_*A*_ varies quasi-linearly, allowing for an accurate determination of the Hill energy through linear interpolation and thus a rapid determination of the *L* value to be used as input in adiabatic simulations. We finally show in the right panel of Figure 3 the variation of the molar Hill energy *L̅* against *P* for both adiabatic and isothermal simulations. The plot reveals that the computed molar Hill energy *L̅*_*I*_ during the isothermal simulations is in very good agreement with the *L*_*A*_ values imposed during the adiabatic simulations, thereby confirming the validity of the proposed grand-isochoric adiabatic simulation approach for mixtures.

**Figure 3:**
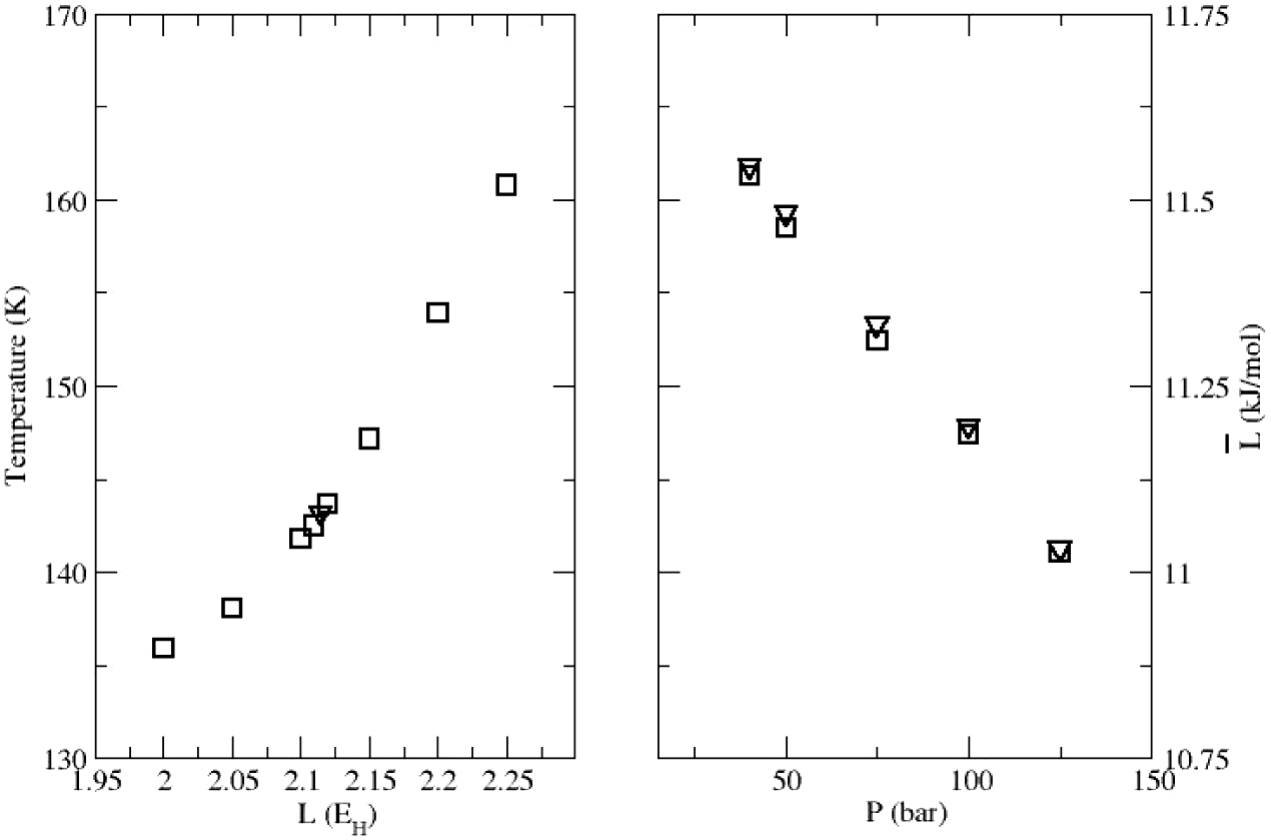
Finding the input value for *L*, i.e., the total Hill energy of the system (here given in Hartree or *E_H_*), when setting up an adiabatic simulation. The left panel shows how to determine its value by varying systematically the input value for *L* in (μ*_Ar_*, *μ_Kr_*, *V*, *L*) simulations (μ_*Ar*_ = −346.95 kJ/kg and μ_*Kr*_ = −206.12 kJ/kg are constant and correspond to the state point (*P* = 40 bar,*T* = 143.15 K) as indicated in Table 2). The value of *L* is identified when the average *T* collected during the adiabatic simulations (shown as squares) coincide with the target temperature of 143.15 K. We also provide for validation purposes the average total Hill energy collected during a grand-canonical simulation for the same sets of chemical potential and *T* = 143.15 K (shown as a circle). The panel on the right shows pairs of matching (*T*, *L*) between adiabatic (open squares) and isothermal (open triangles) simulations for pressures ranging from 40 bar to 125 bar.

Before turning to simulations of the adsorption process, we examine how adiabatic simulations of bulk mixtures already yield faster convergence rates than isothermal simulations. To this end, we study how fast a simulation cell fills up as the thermodynamic conditions approach coexistence, or equivalently, as supersaturation decreases. We define the supersaturation *s* = *P*/P*, where P* = 18.6 bar denotes the coexistence pressure at *T* = 143.15 K for the equimolar mixture. We then compare the filling process at decreasing pressures from *P* = 130 bar to *P* = 118.33 bar, corresponding to superaturations ranging from *s* = 7 to *s* = 6.4, using adiabatic or isothermal simulations. Figure 4 shows the average number of MC steps necessary to reach convergence for (μ_*Ar*_, *μ*_*Kr*_, *V*, *T*) and (μ_*Ar*_, *μ*_*Kr*_, *V*, *L*) simulations. Here, results are obtained by averaging over 50 realizations, with each realization defined as a new set of initial conditions, i.e., here, an empty simulation cell (*N* = 0) and a different seed, and thus sequence, for the generation of the random numbers used in the simulations. The top panels show the average total number of atoms against the number of MC steps for both types of simulations. The results demonstrate that the two types of simulations exhibit markedly different behaviors. Isothermal simulations display a high sensitivity to the degree of supersaturation and converge much more slowly when *s* decreases. On the other hand, adiabatic simulations converge at the same rate for all supersaturations and exhibit a much faster convergence, at least 1 order of magnitude faster than isothermal simulations.

**Figure 4:**
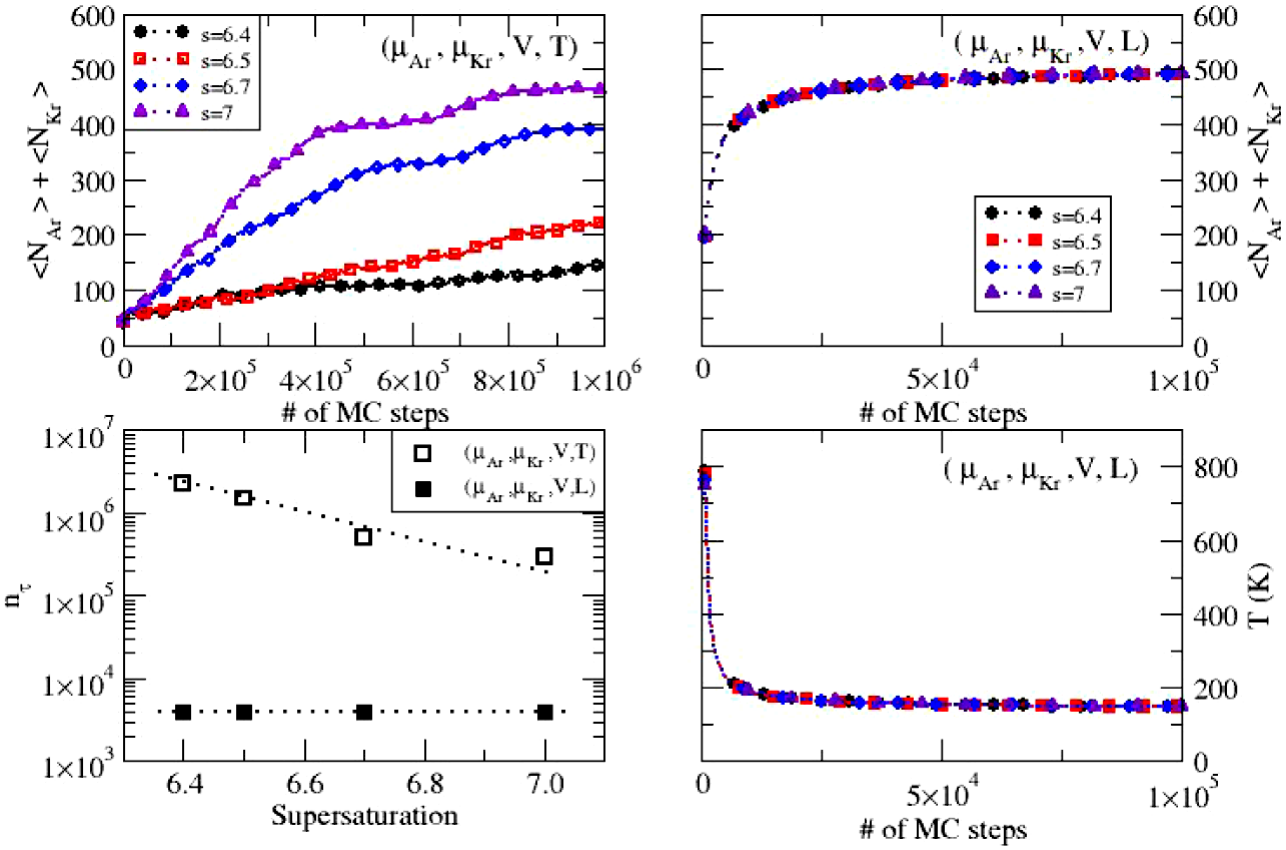
Argon–Krypton mixture for *x_Kr_* = 0.5. (Top panels) Convergence of (μ*_Ar_*, *μ_Kr_*, *V*, *T*) simulations (left) and of the corresponding (μ*_Ar_*, *μ_Kr_*, *V*, *L*) simulations (right) at *T* = 143.15 K and for supersaturations *s* ranging from 6.4 to 7. The results shown here are averaged over 50 realizations. The plots reveal that isothermal simulations converge much more slowly as the supersaturation decreases. (Bottom left panel) Number of steps *n*_τ_ necessary to reach convergence for (μ*_Ar_*, *μ_Kr_*, *V*, *T*) and (μ*_Ar_*, *μ_Kr_*, *V*, *L*) simulations against *s*. (Bottom right panel) Temperature vs the number of MC steps during (μ*_Ar_*, *μ_Kr_*, *V*, *L*) simulations, showing that the accelerated convergence of adiabatic simulation results from the ability of the system to undergo large temperature changes during adiabatic sampling.

For the two types of simulations, we determine *n*_τ_, which denotes the characteristic number of MC steps necessary to reach convergence. To achieve this, we propose the following functional form to model the change in the total number of atoms [⟨*N*_*Ar*_⟩ + ⟨*N*_*Kr*_⟩] as a function of *n*_*MC*_, which corresponds to the number of MC steps,

27 $$\left[\left\langle N_{Ar}\right\rangle +\left\langle N_{Kr}\right\rangle \right]=N_{\infty }\left[1-\exp\left(-\frac{n_{MC}}{n_{\tau }}\right)\right]$$

in which *N*_∞_ denotes the total number of atoms when the simulation has converged. The results obtained for the (μ_*Ar*_, *μ*_*Kr*_, *V*, *L*) and (μ_*Ar*_, *μ*_*Kr*_, *V*, *T*) simulations are shown in the left panel at the bottom of Figure 4 as a function of the supersaturation *s*.

We observe dramatically different *n*_τ_ behaviors for the two types of simulations. In the isothermal case, *n*_τ_ increases as supersaturation decreases. This behavior is consistent with a nucleation-controlled event, in which the system to overcome a free energy barrier as conditions approach coexistence.^1,4,7,126^ This very much differs from the adiabatic case, where *n*_τ_ remains constant over the range of supersaturation considered here. The latter behavior is consistent with the absence of barriers under adiabatic conditions and accounts for the increased efficiency of (μ, *V*, *L*) simulations when conditions become close to coexistence.

What is the underlying reason for the fast convergence of adiabatic simulations? To shed light on this point, we calculate the average temperature during adiabatic simulations (using eq 23) and plot its variation as a function of the number of MC steps in the bottom right panel of Figure 4. We observe that, for all supersaturations examined here, the average temperature exhibits a sharp increase early during the simulations and, initially, largely exceeds the target temperature of *T* = 143.15 K. This corresponds to the system quickly filling up with atoms and thus bypassing the slow, barrier-controlled filling process that takes place in the isothermal simulations. Then, the average temperature decreases and rapidly reaches the equilibrium value of 143.15 K, indicating that the simulations have converged (see also the behavior for the total number of atoms in the system in the top right panel). The efficiency of adiabatic simulations can thus be traced back to the fact that high-temperature/low-potential energy configurations can be efficiently sampled under adiabatic sampling, unlike in isothermal simulations, where temperature is constant and a large free energy barrier has to be overcome at low supersaturations to explore such configurations.

We now turn to the simulation of the adsorption of an equimolar Ar–Kr mixture in an MCM-41 nanopore at *T* = 143.15 K and compare the efficiency of adiabatic and isothermal simulations. To this end, we carry out grand-canonical simulations (all starting from an empty MCM-41 pore) and for a series of (μ_*Ar*_, *μ*_*Kr*_) sets corresponding to pressures ranging from 1 bar to 10 bar of the gaseous Ar–Kr mixture. Then, we carry out grand-isochoric adiabatic simulations, also starting from an empty MCM-41 pore, and for the same series of (μ_*Ar*_, *μ*_*Kr*_) sets. We provide in the Supporting Information the simulation parameters for the 2 series of simulations. The results are shown in Figure 5 and establish that the two simulation methods provide results in excellent agreement over the simulated pressure range. Indeed, both sets of simulations predict the adsorption of a low-density phase for pressures below 5 bar and of a high-density phase for pressures greater than 7 bar (see the total number of atoms adsorbed in MCM-41 in Figure 5a). Looking in more detail at the results, we find that the numbers of atoms adsorbed for each species are also in excellent agreement for the two methods. This confirms that simulations of mixture adsorption in the grand-isochoric ensemble provide a reliable approach to predict the selectivity of an adsorbent toward a specific adsorbate, here Kr. We add that, from a practical standpoint, when the conditions are away from the adsorption step, the number of adsorbed atoms *N*, and thus *L*, vary slowly with *P*. This means that we only need to perform two short trial runs to determine the equation for the *L*–*T* line, determine *L* from this equation to match the target temperature, and then perform a (long) production run for the selected *L* value. Since adiabatic simulations converge very quickly under these conditions (see Figure 7), the two trial runs are much shorter, at least 1 order of magnitude shorter, than the production run. Thus, they do not impact the speed-up and the low-*N* or high-*N* branches of the adsorption isotherms can be computed very efficiently with adiabatic simulations. The mapping requires additional trials in adsorption steps. In such situations, *N* and thus *L* vary rapidly with *P* and we use typically 5 runs to find a suitable *L* interval and perform a linear interpolation. However, this only applies to very few state points, i.e., pressures that fall within the adsorption isotherm step. Here, this reduces to a single pressure *P* = 6 bar in Figure 5a. Figure 5b shows the extent of the fluctuations in the adsorption step, that is, at *P* = 6 bar which corresponds to condensation. More specifically, we show how, during an adiabatic simulation, the number of adsorbed atoms of each type (shown every 10^4^ MC steps) varies, providing insight into adsorption isotherms and selectivities, as well as the potential energy of the adsorbed phase. We also plot the grand-canonical average properties, shown as lines in Figure 5. The plots show that, even through the state point considered here is right in the middle of the adsorption step and of phase-switching, fluctuations are fairly small with standard deviations of 6 atoms for the”instantaneous” numbers of adsorbed atoms of each type. Similarly, we find a very moderate standard deviation for the potential energy of the adsorbed phase. We also show in Figure 5b a plot that indicates that shows the close interdependence between *L* and *N* in the adsorption step, and will return to this point when discussing bimodality and metastability later in this section.

**Figure 5:**
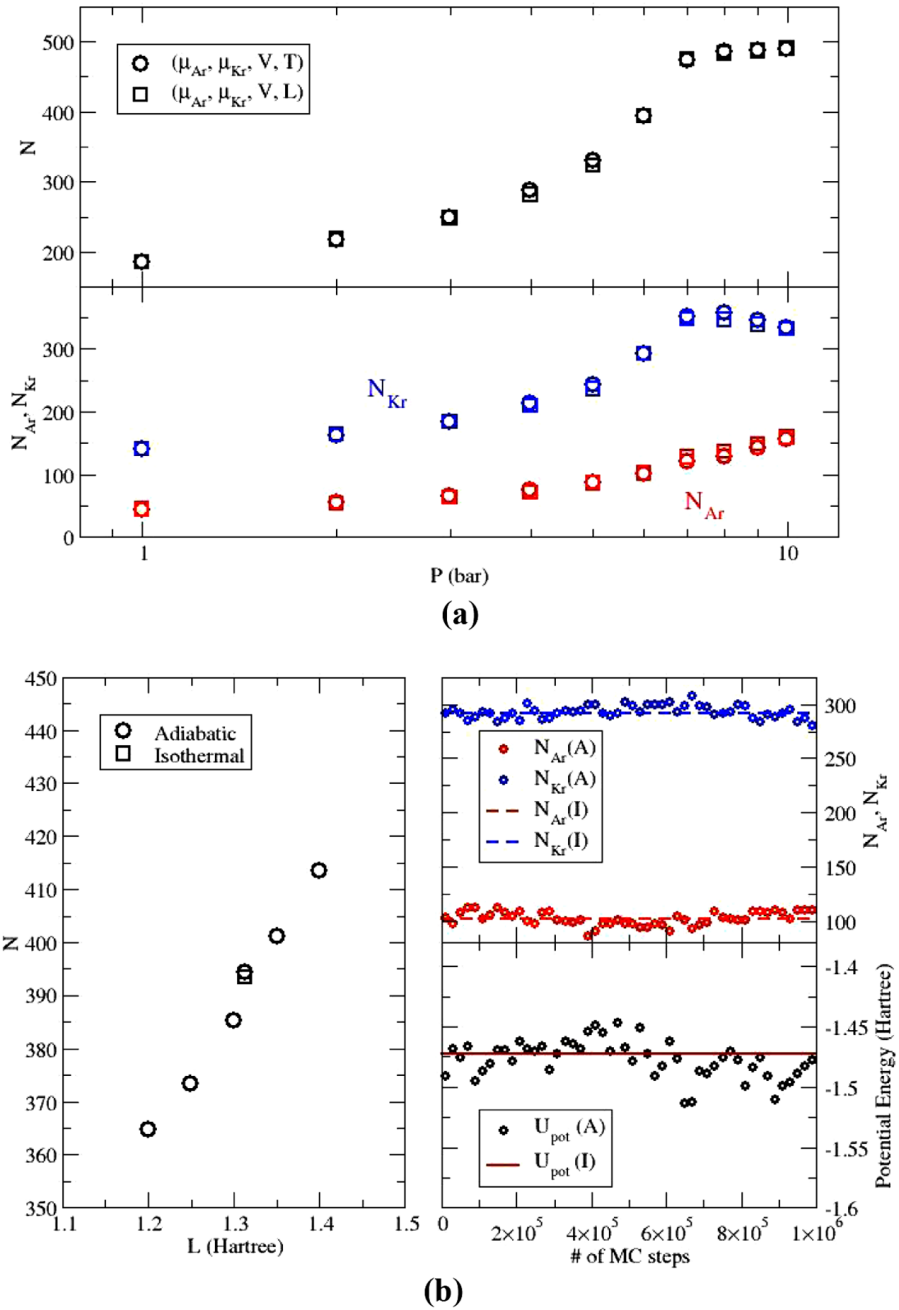
Adsorption of an equimolar Argon–Krypton mixture in MCM-41 at *T* = 143.15 K. (a): (Top) Adsorption isotherm, with *N* denoting the total number of atoms, obtained with isothermal (μ*_Ar_*, μ*_Kr_*, *V*, *T*) simulations and (μ*_Ar_*, *μ_Kr_*, *V*, *T*) simulations. (Bottom) Numbers of Ar atoms adsorbed (in red) and number of Kr atoms adsorbed (in blue) obtained with the two types of simulations. In both plots, isothermal simulations results are shown as circles and adiabatic simulation results as squares. (b): Behavior of adiabatic simulations in the phase-switching region (*P* = 6 bar). The plots show, on the left, the variation of *N* with *L*, together with a comparison with the isothermal (grand-canonical) average, and show, on the right, the extent of the fluctuations in *N_Ar_* and *N_Kr_* (top), and in the potential energy (bottom) when equilibrium has been reached.

Now that we have assessed the validity of grand-isochoric adiabatic simulations for the mixture adsorption, we focus on the comparison between the rates at which isothermal and adiabatic simulations converge. For this purpose, we simulate the adsorption process over a large (50) number of realizations with both methods and collect an average over all realizations of the system’s behavior. Each realization is defined here as a Monte Carlo run that starts with an initially empty MCM-41 pore and a different sequence of random numbers. We show in Figure 6 the average number of adsorbed atoms obtained from isothermal simulations (left panel) and adiabatic simulations (right panel). A rapid inspection of the results shows that, for the three pressures considered in our analysis (*P ∈* (6, 8) bar), the MCM-41 pore fills up much more rapidly with adiabatic simulations than with isothermal simulations. For instance, after 1 × 10^5^ MC steps, the total number of adsorbed atom is, on average, of about 300 for isothermal simulations (see top left panel), while it is already 400 for adiabatic simulations (see top right panel). This conclusion applies to the total number of atoms adsorbed, but also to the number of atoms adsorbed for each species (see bottom plots in Figure 6).

**Figure 6:**
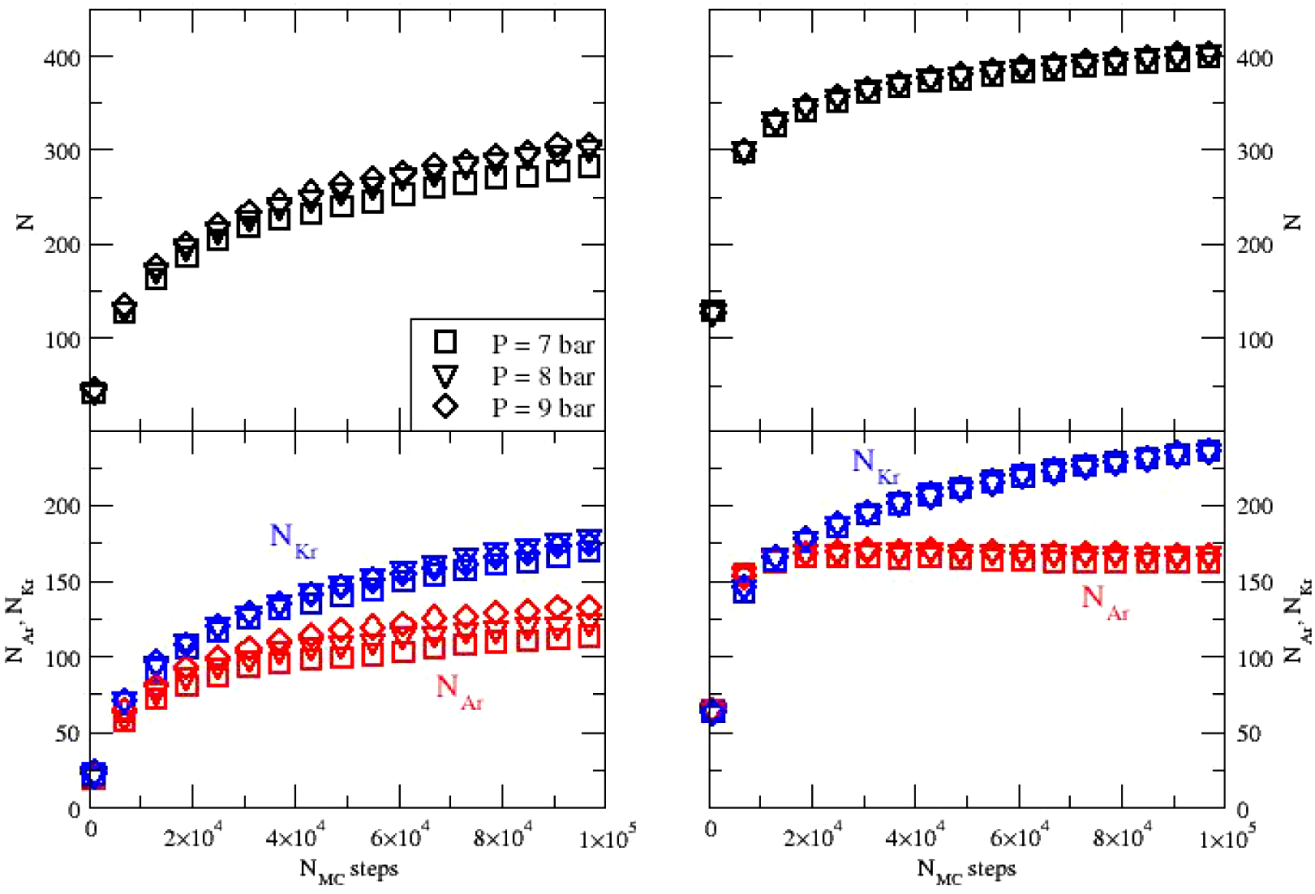
Convergence of isothermal and adiabatic simulations for the adsorption in MCM-41 of an equimolar Ar–Kr mixture at *T* = 143.15 K and for pressures of *P* = 7 bar, *P* = 8 bar, and *P* = 9 bar. The left panel shows the results obtained with isothermal simulations for the total number of atoms adsorbed *N* = *N_Ar_* + *N_Kr_* (top) and for the two components (bottom) as a function of the number of MC steps. The right panel shows the adiabatic simulation results for *N* (top) and for the two components (bottom) against the number of MC steps. For both types of simulations, the results are obtained by averaging over 50 realizations starting from an initially empty MCM-41 pore and a different random numbers sequence.

To analyze this behavior, we examine how temperature varies over 50 realizations of the adiabatic adsorption process and plot the results on the left panel of Figure 7. As with bulk systems, the faster convergence of adiabatic simulations can be attributed to the ability of adiabatic simulations to temporarily raise the temperature of the system during the first few thousand MC steps of the simulations. In turn, this enables adiabatic simulations to visit high energy configurations of the system, which are typically rejected in isothermal simulations because of the low Boltzmann probabilities for these configurations at *T* = 143.15 K. This means that, during the early stages of the adiabatic simulations, the adsorption of atoms via random insertions has a high acceptance rate, unlike in isothermal simulations. Then, as shown in Figure 7, the temperature decays toward the equilibrium value of 143.15 K as the adsorbed phase thermalizes.

**Figure 7:**
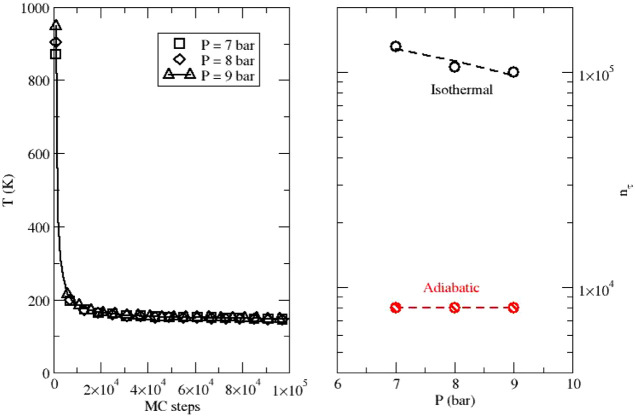
Convergence analysis. (Left) Variation of the temperature of the adsorbed phase during adsorption when simulated adiabatically. Simulation results obtained with (*μ*_*Ar*_, *μ*_*Kr*_, *V*, *L*) simulations for equimolar Ar–Kr mixtures at *T* = 143.15 K and for pressures *P* = 6 bar, *P* = 7 bar, and *P* = 8 bar. (Right) Convergence, measured as *n*_τ_, of (*μ*_*Ar*_, *μ*_*Kr*_, *V*, *T*) simulations and (*μ*_*Ar*_, *μ*_*Kr*_, *V*, *L*) simulations against pressure. With increasing pressure (or supersaturation), the isothermal simulations converge faster. On the other hand, the adiabatic simulations exhibit a constant rate of convergence, that is notably faster than with isothermal simulations for all pressures.

To quantitatively compare the rate of convergence for the two methods, we use the same metric as for bulk simulations and determine the characteristic number of MC steps *n*_τ_ necessary to reach convergence (see eq 27). We show the results obtained with both simulation methods on the right panel of Figure 7. We observe two different behaviors. In the case of isothermal simulations, *n*_τ_ is found to decrease with *P* or, equivalently, as the supersaturation increases. This behavior is in line with what is expected at fixed temperature, i.e., when the system has to overcome a free energy barrier to complete the transition from a low-density adsorbed phase to a high-density adsorbed phase. On the other hand, in the case of adiabatic simulations, *n*_τ_ remains constant for the three pressures and supersaturation considered in the analysis. These findings are consistent with those obtained for the bulk. Very importantly, the *n*_τ_ values for the adiabatic simulations are found to be 1–2 orders of magnitude smaller than for isothermal simulations, indicating that adiabatic simulations exhibit a speed-up of 1–2 orders of magnitude to convergence. This suggests that adiabatic simulations could be very efficient for the computational screening large libraries of nanoporous and mesoporous structures.

Now that we have validated the grand-isochoric adiabatic simulation approach for the adsorption of mixtures and established its efficiency when compared to isothermal simulations, we turn to a more challenging case for which metastability has greater impact on the adsorption process and bimodality starts to develop. To this end^32,36^ we simulate the adsorption of the Ar–Kr mixture with *x*_*Ar*_ = 0.5 in a longer MCM-41 pore with a length of 100 Å rather than 40 Å. We also perform the simulations at a lower temperature of *T* = 120 K rather than *T* = 143.16 K. We provide in Figure 8 the results obtained during long grand-canonical Monte Carlo runs. The top panel shows the variation of the number of atoms adsorbed in the pore for P = 1.1, 1.15, 1.2 and 1.5 bar. At *P* = 1.1 bar and *P* = 1.5 bar, the system does not exhibit any sign of metastability and converges rapidly, i.e., within 10^6^ MC steps, toward the lower branch (for *P* = 1.1 bar) and the higher branch (for *P* = 1.5 bar) of the adsorption isotherm. On the other hand, results for the two intermediate pressures show that metastability impacts the adsorption process. Results at *P* = 1.15 bar most particularly start to exhibit signs of bistability with the onset of two adsorption plateaus. After the initial rapid increase in the number of atoms adsorbed from 0 to 850 over the first 10^6^ MC steps, the plot shows that this number remains around 850 over the next 3 × 10^6^ MC steps before transitioning and reaching another plateau after 7 × 10^6^ MC steps, indicating that the system has reached the high branch. A similar, however much less pronounced effect, can also be observed at *P* = 1.2 bar and points to the end of the metastability window. What is most interesting, however, is the behavior of the Hill energy during these isothermal simulations of the adsorption process. Looking at the plot of *L* vs number of MC steps in Figure 8 for *P* = 1.15 bar, we find that the behavior observed for *L* mirrors that observed for *N*. This means that, during an isothermal simulation of adsorption and for conditions are within the metastability window, bistability in *N* occurs concomitantly with bistability in *L*. We had shown in Figure 7 how adiabatic simulations achieved faster convergence through large initial temperature changes, but the observation of the concomitant *L* and *N* bistability in isothermal simulations explains why adiabatic simulations are much more efficient. Indeed, isothermal simulations can remain stuck at a metastable low *L* value, i.e., below the equilibrium value of *L* under the adsorption conditions. On the other hand, since adiabatic simulations are carried out at the equilibrium value of *L*, *N* will reach directly its correct (equilibrium) value, given the close interdependence between the two quantities, thereby accounting for the increased efficiency of adiabatic simulations.

**Figure 8:**
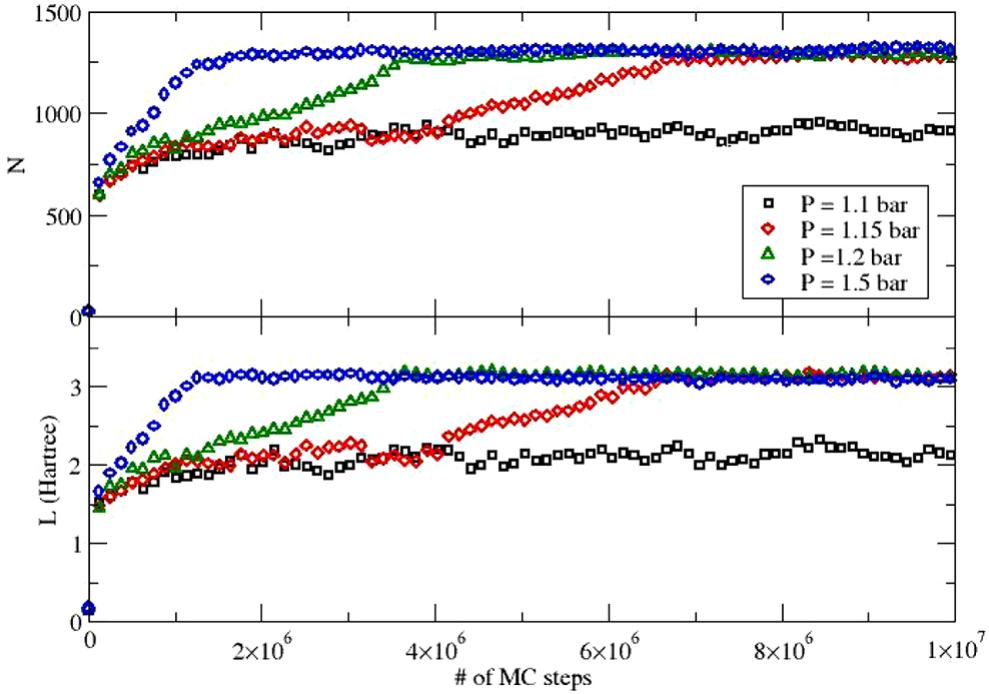
Grand-canonical (isothermal) simulations of adsorption at 120 K and for a longer MCM-41 pore of 100 Å. Results are shown for pressures ranging from 1.1 bar to 1.5 bar and indicate the onset of concomitant bistability at *P* = 1.15 bar in both the *N* vs # of MC steps (Top) and *L* vs # of MC steps (Bottom) plots. The close interdependence of *N* and *L* also reveals why isothermal simulations can remain stuck at the lower (metastable) value of *L* for a long time converge much more slowly than adiabatic simulations, which are carried out at the higher (equilibrium) value of *L* reach directly the equilibrium plateau for N.

## Conclusions

In this work, we examine how adiabatic simulations can alleviate convergence slowdowns that arise in isothermal simulations due to metastability and the existence of large free energy barriers when temperature is constant. We focus on the case of mixtures and perform simulations of bulk phases and adsorption processes. To this end, we extend the grand-isochoric adiabatic simulation method, which had only been applied to simulate single-component systems so far, to multicomponent systems and determine the acceptance rules necessary to implement a simulation based on this ensemble in a Monte Carlo framework. The grand-isochoric adiabatic ensemble allows for the number of atoms of each species, as well as temperature, to vary while maintaining the volume of the system constant. It is thus the adiabatic counterpart of the well-established (isothermal) grand-canonical ensemble. To assess the reliability of the multicomponent adiabatic simulation approach we propose in this work, we carefully compare the simulation results obtained with the new method against those provided by the isothermal grand-canonical simulation approach on the examples of bulk Ar–Kr liquids and Ar–Kr mixtures adsorbed in an MCM-41 pore. For both bulk and adsorption processes, we obtain excellent agreement between the results obtained with the two approaches for a wide range of thermodynamic properties. This demonstrates the reliability of the grand-isochoric adiabatic simulation method for the prediction of mixture properties and mixture adsorption. Interestingly, as thermodynamic conditions approach coexistence and supersaturation decreases, the number of MC steps necessary to reach convergence increases sharply in isothermal simulations, while adiabatic simulations exhibit the same rate of convergence across all supersaturation values simulated. We also show that adiabatic simulations achieve faster convergence by temporarily raising the system’s temperature well above its equilibrium temperature during the early stages of the simulation. This, in turn, accelerates the exploration of the configuration space, e.g., favoring rapid adsorption, and avoids the slowdown associated with the large free energy barriers isothermal simulations encounter. Overall, for both bulk and nanoconfined systems, we observe a speed-up between 1 and 2 orders of magnitude when adiabatic simulations are used. Given the recent interest in the automated exploration of large combinatorial spaces in materials science and, more specifically, in the assessment of large libraries of hypothetical nanoporous materials involved in gas storage and separation processes, the grand-isochoric adiabatic simulation method is a promising approach for the rapid and efficient screening of materials for adsorption and separation processes.

## Supplementary Material



## Notes

Data tables for the figures and trends discussed in the Results section (PDF)
