# Supplementary material for: Rapid Exploration of Mixture Adsorption via Adiabatic Sampling
Source: J Phys Chem C Nanomater Interfaces. 2026 Jun 17;130(26):9180–94. doi: 10.1021/acs.jpcc.6c01337 (PMC13339766; doi:10.1021/acs.jpcc.6c01337)
Supplement: Supplementary file 1 [file jp6c01337_si_001.pdf]

SI: Rapid Exploration of Mixture Adsorption via Adiabatic Sampling

Caroline Desgranges*,† and Jerome Delhommelle*,‡

†*Department of Physics and Applied Physics, University of Massachusetts, Lowell, MA 01854, USA*

‡*Department of Chemistry, University of Massachusetts, Lowell, MA 01854, USA*

E-mail: caroline_desgranges@uml.edu; jerome_delhommelle@uml.edu

Phone: 978-934-3721; 978-934-4367

Table S1: Argon at vapor-liquid coexistence: Molar Hill energy for the liquid and vapor phases extracted from experimental data (Exp.)[1] and previous simulation work (Sim.)[2]

T (K)	L_{liq} (Exp.) (kJ/mol)	L_{vap} (Exp.) (kJ/mol)	L_{liq} (Sim.) (kJ/mol)	L_{vap} (Sim.) (kJ/mol)
85	4.64	10.39	–	–
90	5.11	10.70	4.95	11.30
95	5.60	11.02	–	–
100	6.11	11.35	6.01	11.70
105	6.64	11.67	–	–
110	7.19	12.00	7.07	12.17
115	7.75	12.32	–	–
120	8.33	12.64	8.19	12.77
125	8.93	12.94	–	–
130	9.56	13.24	9.39	13.39
135	10.22	13.51	–	–
140	10.89	13.73	10.81	13.85
145	11.59	13.88	–	–
150	12.65	13.69	–	–

Table S2: Equimolar Ar-Kr mixture at coexistence[2] for $T = 143.15$ K: pressure dependence of Hill energies for the liquid and vapor phases (see Fig. 1 in the paper).

P (bar)	L_{liq} (kJ/mol)	L_{vap} (kJ/mol)
6	11.08	17.99
8	11.28	17.59
9	11.33	17.39
11	11.44	17.01
15	11.61	16.36
18	11.66	15.95
21	11.70	15.59
24	11.67	15.28
26	11.64	15.06

Table S3: Simulation parameters for equimolar liquid Ar-Kr mixtures at $T = 143.15$ K and for pressures ranging from 40 bar to 125 bar.

P (bar)	μ_{Ar} (kJ/kg)	μ_{Kr} (kJ/kg)	L (E_H)
40	-346.95	-206.12	2.116
50	-345.90	-205.70	2.117
75	-343.52	-204.70	2.112
100	-341.14	-203.71	2.106
125	-338.91	-202.65	2.098

Table S4: Equimolar liquid Ar-Kr mixtures at T = 143.15 K and pressures ranging from 40 bar to 125 bar: comparison between simulation results obtained using either isothermal $(\mu_{Ar}, \mu_{Kr}, V, T)$ simulations (denoted by I) or adiabatic $(\mu_{Ar}, \mu_{Kr}, V, L)$ simulations (denoted by A).

P (bar)	$x_{Ar,I}$	U_I (kJ/mol)	N_I	T_A (K)	$x_{Ar,A}$	U_A (kJ/mol)	N_A
40	0.50	-5.82	480.8	143.2	0.50	-5.84	481.7
50	0.50	-5.84	482.5	143.7	0.50	-5.86	484.8
75	0.50	-5.90	487.5	143.6	0.50	-5.93	490.2
100	0.50	-5.93	491.8	144.0	0.50	-5.95	494.2
125	0.50	-6.03	498.4	143.6	0.50	-6.02	499.4

Table S5: Equimolar liquid Ar-Kr mixtures for at T = 143.15 K and pressures ranging from 40 bar to 125 bar: comparison between the molar Hill free energies obtained using either isothermal $(\mu_{Ar}, \mu_{Kr}, V, T)$ simulations (denoted by I) or adiabatic $(\mu_{Ar}, \mu_{Kr}, V, L)$ simulations (denoted by A).

Pressure (bar)	L_A (kJ/mol)	L_I (kJ/mol)
40	11.53	11.54
50	11.46	11.48
75	11.31	11.33
100	11.19	11.20
125	11.03	11.03

Table S6: Number of MC steps n_τ necessary to reach convergence for 50 realizations of isothermal (denoted by I) and adiabatic (denoted by A) simulations of equimolar Ar-Kr liquid mixtures for different supersaturations.

Supersaturation	$n_{\tau,I}$	$n_{\tau,A}$
6.4	2.31×10^6	4×10^3
6.5	1.51×10^6	4×10^3
6.7	5.04×10^5	4×10^3
7.0	2.91×10^5	4×10^3

Table S7: Adsorption of equimolar Ar-Kr mixtures in MCM-41 at $T = 143.15$ K: comparison between simulation results obtained with isothermal $(\mu_{Ar}, \mu_{Kr}, V, T)$ simulations (denoted by I) and adiabatic $(\mu_{Ar}, \mu_{Kr}, V, L)$ simulations (denoted by A).

P (bar)	μ_{Ar} (kJ/kg)	μ_{Kr} (kJ/kg)	N_I	$N_{Ar,I}$	$N_{Kr,I}$	L_A (E_H)	T_A (K)	N_I	$N_{Ar,I}$	$N_{Kr,I}$
1	-446.21	-229.36	186	45	141	0.68	143.0	186	45	141
2	-425.81	-219.69	218	55	163	0.74	143.3	218	54	164
3	-413.93	-214.10	249	65	184	0.82	143.3	249	64	185
4	-405.57	-210.20	288	75	213	0.93	142.7	282	72	210
5	-399.11	-207.22	330	86	244	1.07	143.3	323	86	237
6	-393.9	-204.85	393	101	292	1.31	143,6	394	102	292
7	-389.49	-202.86	474	122	352	1.59	143.2	476	128	348
8	-383.9	-202.21	486	128	358	1.59	142.8	483	136	347
9	-377.94	-202.43	487	142	345	1.61	143.8	487	148	339
10	-373.20	-202.73	490	157	333	1.61	143.5	491	159	333

Table S8: Number of MC steps n_τ necessary to reach convergence for 50 realizations of isothermal (denoted by I) and adiabatic (denoted by A) simulations of the adsorption of equimolar Ar-Kr mixtures in MCM-41 for different pressures.

P	$n_{\tau,I}$	$n_{\tau,A}$
7	1.31×10^5	8×10^3
8	1.05×10^5	8×10^3
9	1×10^5	8×10^3

References

(1) Vargaftik, N. B.; Vinoradov, Y. K.; Yargin, V. S. *Handbook of Physical Properties of Liquids and Gases*; Begell House, New York, 1996.

(2) Desgranges, C.; Delhommelle, J. Many-body effects on the thermodynamics of fluids, mixtures, and nanoconfined fluids. *J. Chem. Theory Comput.* **2015**, *11*, 5401–5414.
